# Endometriosis Might Be Inversely Associated with Developing Chronic Kidney Disease: A Population-Based Cohort Study in Taiwan

**DOI:** 10.3390/ijms17071079

**Published:** 2016-07-07

**Authors:** Ben-Shian Huang, Wen-Hsun Chang, Kuan-Chin Wang, Nicole Huang, Chao-Yu Guo, Yiing-Jen Chou, Hsin-Yi Huang, Tzeng-Ji Chen, Wen-Ling Lee, Peng-Hui Wang

**Affiliations:** 1 Department of Obstetrics and Gynecology, Yang-Ming University Hospital, Ilan 260, Taiwan; benshianhuang@gmail.com; 2 Department of Obstetrics and Gynecology, National Yang-Ming University, Taipei 112, Taiwan; 3 Department of Nursing, Taipei Veterans General Hospital, Taipei 112, Taiwan; whchang@vghtpe.gov.tw; 4 Department of Obstetrics and Gynecology, Taipei Veterans General Hospital, Taipei 112, Taiwan; 5 Department of Nursing, Oriental Institute of Technology, New Taipei City 220, Taiwan; kcw@mail.oit.edu.tw; 6 Institute of Public Health, National Yang-Ming University, Taipei 112, Taiwan; syhuang@ym.edu.tw (N.H.); cyguo@ym.edu.tw (C.-Y.G.); yjchou@ym.edu.tw (Y.-J.C.); 7 Biostatics Task Force, Department of Medical Research and Education, Taipei Veterans General Hospital, Taipei 112, Taiwan; sweethsin509@gmail.com; 8 Department of Family Medicine, Taipei Veterans General Hospital, Taipei 112, Taiwan; tjchen@vghtpe.gov.tw; 9 Department of Medicine, Cheng-Hsin General Hospital, Taipei 112, Taiwan; 10 Department of Medical Research, China Medical University Hospital, Taichung 440, Taiwan

**Keywords:** chronic kidney disease, cohort study, endometriosis, epidemiology, gender, menopause

## Abstract

This study was conducted to determine the risk of chronic kidney disease (CKD) among women with endometriosis in Taiwan. We conducted a retrospective cohort study using the National Health Insurance Research Database of Taiwan. A total of 27,973 women with a diagnosis of endometriosis and 27,973 multivariable-matched controls (1:1) from 2000 to 2010 were selected. Cox regression and computed hazard ratios (HR) with 95% confidence intervals (95% CI) were used to determine the risk of CKD among women with endometriosis. The incidence rates (IR, per 10,000 person-years) of CKD among women with and without endometriosis were 4.64 and 7.01, respectively, with a significantly decreased risk of CKD (crude HR 0.65, 95% CI 0.53–0.81; adjusted HR 0.69, 95% CI 0.56–0.86) among women with endometriosis. The IR of CKD progressively increased with age, but the trend of lower CKD risk among women with endometriosis was consistent. However, the lower risk of CKD in women with endometriosis was no longer statistically significant after adjusting for menopausal status (adjusted HR 0.85, 95% CI 0.65–1.10). The results suggest that endometriosis is inversely associated with CKD, but this effect was mediated by menopause. The possible mechanism of this association is worthy of further evaluation.

## 1. Introduction

Chronic kidney disease (CKD) is defined as decreased kidney function (glomerular filtration rate (GFR) lower than 60 mL/min per 1.73 m^2^) or kidney damage (i.e., albuminuria) for three months or longer, and there has been a classification scheme based on GFR [1]. The age-standardized global prevalence of CKD stages 1–5 was 10.4% in men (95% confidence interval 9.3%–11.9%) and 11.8% in women (11.2%–12.6%) in adults aged 20 and older [2]. CKD is associated with several risks, such as anemia, diabetes mellitus (DM), and cardiovascular disease (CVD) [3]. A total of 956,200 deaths worldwide were directly associated with CKD in 2013, which represents a 134.6% increase from 1990 in the Global Burden of Disease Study 2013 [4]. Medical costs for treating CKD and end-stage renal disease (ESRD) are tremendous and represent a gigantic financial burden to families and society [5]. DM and CVD are the leading causes of CKD, and environmental exposures, the aging of the population, as well as changes in lifestyle risk factors have all probably contributed to the increased burden of CKD [6]. Additional risk factors for CKD include obesity, tobacco use, high low-density lipoprotein cholesterol, poor diet, and physical inactivity [7].

The gender difference in the incidence and severity of CKD is well documented, and women, especially those who are premenopausal, possess a significantly lower risk of CKD than men. However, in postmenopausal women, the renal function of may be exacerbated, and this contributes to the higher prevalence of CKD in women compared with men [2]. Moreover, at all ages and all estimated GFR strata, the lifetime risk of ESRD is consistently higher for men than for women. Thus, the male gender is an independent risk factor for the development and progression of CKD, and, regardless of previous renal status, men progress to ESRD from CKD more rapidly than premenopausal women [8].

Endometriosis is an estrogen-dependent inflammatory disease, which affects 6%–10% of women of reproductive age [9,10,11]. This disease is defined as the presence of endometrial glandular and stromal tissues in sites outside the uterine cavity, primarily on ovaries and the pelvic peritoneum. Women with endometriosis may suffer from dyspareunia, chronic pelvic pain, infertility, or a combination of these [12]. The classification of endometriosis is evolving from a local disorder to a complex and chronic systemic disease with insights into the molecular and cellular mechanisms.

The association between endometriosis and CKD is not clear, and there are few case reports of endometriosis-related obstructive uropathy [13,14,15,16]. Further, there is no epidemiologic prevalence study of CKD among women with endometriosis. To date, it has been documented that many chronic diseases, such as DM, CVD, rheumatic disease, and chronic liver disease, might be associated with the development of CKD [7].

Our goal is to determine whether endometriosis was associated with CKD after adjusting for the aforementioned factors. To accomplish the purpose, a large-scale, nationwide, controlled cohort study was conducted using the National Health Insurance Research Database of Taiwan.

## 2. Results

### 2.1. General Characteristics

Among the entire cohort of 55,964 women, there were 628,337 total person-years of follow-up, including 314,514 from women with endometriosis and 313,823 from women without endometriosis. From 2000 to 2010, 366 women were diagnosed with CKD. Compared with women without endometriosis, women with endometriosis had higher rates of comorbid infertility, pelvic inflammatory disease (PID), chronic liver disease, CVD, and rheumatoid arthritis (all *p* < 0.0005). In DM between the groups, there was no statistically significant difference (Table 1).

In addition, we used the same database to compare the risk of CKD between men and women. The prevalence of CKD was greater among women than among men (total number of patients with CKD, *n* = 5636; male vs. female = 2733 (48.49%) vs. 2903 (51.51%), *p* = 0.033) (data not shown).

### 2.2. Incidence Rates and Crude and Adjusted Risks of CKD among Women with and without Endometriosis

Among women with and without endometriosis, the incidence rates (IRs) of CKD were 4.64 and 7.01 per 10,000 person-years, yielding a crude hazard ratio (HR) of 0.65 (95% confidence interval (CI) 0.53–0.81, *p* < 0.001); this suggested that there was a lower risk of CKD among women with endometriosis. After adjusting for confounders (menopausal status was not included), there was still a lower risk of CKD among the women with endometriosis (adjusted HR1 0.69, 95% CI 0.56–0.86, *p* < 0.001). However, compared with controls, the significantly lower risk of CKD among women with endometriosis was no longer present when the model was further adjusted for menopausal status (adjusted HR2 0.85, 95% CI 0.65–1.10) (Table 2).

### 2.3. The Role of Age in the Relevance between CKD and Endometriosis

To clarify the role of age in the relevance between CKD and endometriosis, subgroup analysis based on age was conducted using five age groups (those <40, 40–49, 50–59, 60–69 and ≥70 years). With age, the risks of CKD among women with endometriosis significantly increased. The IR of CKD ranged from the lowest IR of 1.32 per 10,000 person-years at age <40 years to the highest IR of 13.34 at age ≥70 years among women with endometriosis (Table 3). In the crude model, we used the youngest group (women <40 years) as the reference, and the HRs (95% CI) among women with endometriosis aged 40–49, 50–59, 60–69, and ≥70 years were 4.72 (95% CI 2.74–8.13), 5.99 (95% CI 3.44–10.44), 9.26 (95% CI 4.85–17.68), and 10.66 (95% CI 4.20–27.06), respectively (*p* < 0.0001). After adjusting for confounders (menopausal status was excluded), the adjusted HR1s (95% CI) of women with endometriosis aged 40–49, 50–59, 60–69, and ≥70 years were 2.86 (95% CI 1.58–5.16), 2.33 (95% CI 1.24–4.37), 2.43 (95% CI 1.16–5.07), and 2.29 (95% CI 0.84–6.23), respectively (*p* = 0.0162). After adjusting for confounders and menopausal status, there was still a constantly higher risk of CKD in older women with endometriosis (adjusted HR2 2.92, 95% CI 1.61–5.27 at 40–49 years; adjusted HR2 2.53, 95% CI 1.34–4.79 at age 50–59 years; adjusted HR2 2.64, 95% CI 1.26–5.54 at age 60–69 years; and adjusted HR2 2.46, 95% CI 0.90–6.73 at age ≥70 years). All analyses revealed that the risk of CKD significantly increased with age among women with endometriosis (Table 3).

The positive correlation between the risk of CKD and age, which was also present among controls (IR of CKD in this population ranged from 2.12 to 24.18 per 10,000 person-years separated by the different age groups) (Table 4), and this suggested that age was the most important and independent risk factor for the development of CKD among women, regardless of endometriosis or not.

### 2.4. Comparison of the Risk of CKD among Women with and without Endometriosis, According to Age

Although the IR of CKD increased with age in women regardless of whether they had endometriosis, it seemed that women with endometriosis possessed a lower risk of CKD than controls, with the crude HRs ranging from 0.53 to 0.73 in the different age groups (Table 4). The trend of lowered CKD risk among women with endometriosis was relatively constant, even after adjusting for confounders (menopausal status was excluded). The adjusted HR1s ranged from 0.52 to 0.74. The lowest risk of CKD among women with endometriosis was found among those aged 40–49 years, with a crude HR of 0.67 (95% CI 0.48–0.95) and an adjusted HR1 of 0.66 (95% CI 0.46–0.94). Similar to the findings shown in Table 2, menopausal status might play an important role in the risk of CKD. After adjusting for confounders and menopausal status, the trend of lowered CKD risk among women with endometriosis appeared to no longer be significant (Table 4).

### 2.5. Comparison of CKD Risk among Women with and without Endometriosis, According to Age

Finally, the time interval between enrollment in each cohort and the diagnosis of newly developed CKD (exposure time or surveillance time) was calculated to evaluate the duration before the patients in this cohort were diagnosed with CKD. The median time for all women with CKD was 6.47 (range 0–15.29) years (Table 5). The median time among women with endometriosis was 7.45 (range 0.12–15.29) years, compared with 5.86 (range 0–14.97) years among women without endometriosis, which reached statistical significance (*p* = 0.0002). By contrast, the median age of the women with endometriosis who were diagnosed with CKD was 49.5 (range 19–81) years, which was very similar to that of women without endometriosis (median 49.5 years, range 20–82 years), and this did not differ significantly.

These data suggest that women with endometriosis have a lower risk of CKD in Taiwan. This lower risk is supported by the longer interval between enrollment in the cohort and the diagnosis of CKD among women with endometriosis, but this is not biased by surveillance in the entire cohort (Table 6).

## 3. Discussion

Our study indicated that there was a lower risk of CKD among women with endometriosis compared with women without endometriosis (crude HR 0.65, 95% CI 0.53–0.81, *p* < 0.001). Much comorbidity is associated with CKD. For example, CVD and DM are the well-known leading causes of CKD, and this is true both worldwide and in Taiwan [7,17,18,19]. DM is the first leading cause of CKD, and chronic glomerulonephritis and CVD are the second and third leading causes of CKD in Taiwan [7,18,19]. Other factors, such as autoimmune disease, systemic infection, and urinary tract infection, have been reported to be an associated with CKD [17]. Therefore, these factors should be adjusted for in the estimation of CKD in the population. Except for DM, we found that women with endometriosis possessed a higher rate of these unfavorable comorbidities, including PID, infertility, CVD, chronic liver disease, and rheumatoid arthritis compared with those without endometriosis. Adjusting for these confounders to estimate the risk of CKD among women with endometriosis, women with endometriosis still possessed a consistently lower risk of CKD than did those without endometriosis (adjusted HR1 0.69, 95% CI 0.56–0.86, *p* < 0.001).

In addition to comorbidities, age is a very important factor for many acute and chronic diseases, including CKD [20]. Our study further confirmed the important role of age in CKD. The IR of CKD was lowest among women <40 years, at 1.32 and 2.12 for those with and without endometriosis, respectively, and showed a progressive and significant increase, with the highest IRs of CKD found for women age ≥70 years, at 13.34 and 24.18 for women with and without endometriosis, respectively. Consistent with the lower overall risk of CKD among women with endometriosis compared with those without endometriosis, women with endometriosis possessed a relatively consistent lower risk or a trend towards a lower risk of CKD compared with women without endometriosis in the different age subgroups. We also found that the lowest risk of CKD among women with endometriosis compared with those without endometriosis was for women aged 40–49 years, and this significantly decreased risk of CKD among women with endometriosis lessened among women ≥50 years of age, suggesting that 50 years might be an important age at which to check for the development of CKD.

In Taiwan, the average age at menopause is 50 years [21,22,23,24]. Menopause, defined by ceased menstruation for 6 cycles without pregnancy, is mainly secondary to estrogen deficiency [25,26,27]. Although endometriosis and CKD appear to be different diseases, it is evident that the contribution of sex hormones, especially estrogen, as a probable key factor in the development of them both [8,9,10,11,12,18,22]. In fact, attention has recently been paid to understanding the role of estrogen, mediated by the action of estrogen receptors (at least 3 distinct ERs, i.e., ER-α, ER-β, and GPER (known as G-protein-couple protein 30)) either directly or indirectly [18,28], as they are related to the physiological, pathological, and pathophysiological features of many human systems, such as the reproductive organs, the genito-urinary system, the cardiovascular system, and the neurological and skeleton systems [8,28,29,30,31,32]. Although the pathogenesis of endometriosis is complex, this disorder is believed to be secondary to aberrations of estrogen production and function [8,9,10,11,12,31]. Bulun and colleagues found that defective CpG methylation affecting several genes that encode key transcription factors, including ER-β, steroidogenic factor-1 (SF-1), and GATA6 in endometriosis leads to the overproduction of local estrogen and prostaglandins and the suppression of progesterone receptor [31].

Further evidence to support the possible role of estrogen in the development of CKD can be tested by gender differences. Male gender is well documented as an independent risk factor for the development and progression of CKD [8,32]. However, the steep decline in flow-mediated vasodilatation in women starts at menopause, and the deterioration of renal function in women, compared with men, seems to be more severe and faster [8,33,34]. This suggests that gender difference appears to affect the incidence and severity of these diseases, and, especially after menopause, women possess a significantly higher risk for developing many physiologically and pathologically related health problems, such as CVD, bone loss, and CKD.

To clarify the different risk of CKD in both genders, we used the same database to compare the risk of CKD between men and women. It was relatively surprising to find that the prevalence of CKD in Taiwan is greater among women than among men (total number of patients with CKD, *n* = 5636; male vs. female = 2733 (48.49%) vs. 2903 (51.51%), *p* = 0.033), and this finding does not support the concept that the lifetime risk of ESRD for men is consistently higher at all ages and all estimated GFR strata than for women [35,36]. The odds ratio (OR) of CKD was 1.06 (95% CI 1.01–1.12, *p* = 0.033) for women among all age groups. No statistically significant difference between women and men was revealed when considering those ≤50 years of age (OR 0.99, 95% CI 0.89–1.11, *p* = 0.883). As expected, women aged >50 years had a much higher risk of CKD than men aged >50 years (OR 11.77, 95% CI 11.05–12.54, *p* < 0.0001), suggesting that menopause is indeed a strong risk factor for the development of CKD in women. This supports our findings that, after adjusting for menopausal status, the lower risk of CKD among women with endometriosis was not significant compared with that of women without endometriosis.

How does estrogen mediate renal protection? Several animal models of renal diseases have revealed that the progression of renal injury in female animals is slower than in their male littermates, and both estrogen substitution and orchiectomy can slow disease progression in male rats. Estrogen can restrain collagen synthesis in glomerular mesangial cells by attenuating the expression of transcription factor AP-1 and the activity of angiotensin II-induced mitogen-activated protein kinase (MAPK). Proteinuria and glomerular fibrosis after experimental renal damage can be reduced by estrogen application, including in ischemia-reperfusion animal models [37,38]. Nevertheless, the contributory mechanisms of estrogen are not clear, while it has been proposed that ER-α may mediate the effect of estrogen-induced renal and possibly cardiovascular protection [37]. Kummer et al. found that estrogen, mediated through ER-α, could protect podocytes from apoptosis and subsequent glomerulosclerosis [38]. Diwan et al. conducted one adenine-fed rat model, and there was a significantly lower ER-α expression in male rat kidneys than in female rat kidneys. The results revealed that female adenine-fed rats possessed significantly less kidney functional decrease than male adenine-fed rats, but, between the two groups, there were similar CKD-related molecular changes, including ERK 1/2, hemeoxygenase-1, and tumor necrosis factor-α (TNF-α) [37]. Compared with female rats, the decreased expression of ER-α in male rat kidneys and changes in plasma testosterone and estrogen concentrations in male rats may be related to this increased renal damage; all of this suggests the favorable role of ER-α in the kidney [34]. However, the effect of long-term and continuous estrogen use on renal function in postmenopausal women remains controversial. Both a 10-year prospective study and a cross-sectional of postmenopausal estrogen therapy and renal function showed that there was a better GFR in estrogen users than in non-users, although the prospective study showed no differences in GFR by estrogen use and a decreased urine albumin-to-creatinine ratio among mostly long-term current users after 10 years of follow-up [39].

The most important strength of the current study is that it might be the first nationwide, population-based study in an Asian country. In addition, using this national population-based study, we further confirmed that CKD is an age-dependent disease. Third, the prevalence of CKD is much higher in women than that in men, and postmenopausal women had a much higher risk of CKD than age-matched men. However, this study has some limitations. First, we did not classify CKD by GFR, and we also did not classify the severity of endometriosis based on American Society of Reproductive Medicine stage [40]. Second, we did not evaluate the effect of medication or surgical intervention, including oral pills, non-steroid anti-inflammatory drugs, gonadotropin-releasing hormone agonist, and other agents, which may influence the risk estimation.

## 4. Materials and Methods

### 4.1. Study Population

**Figure 1:**
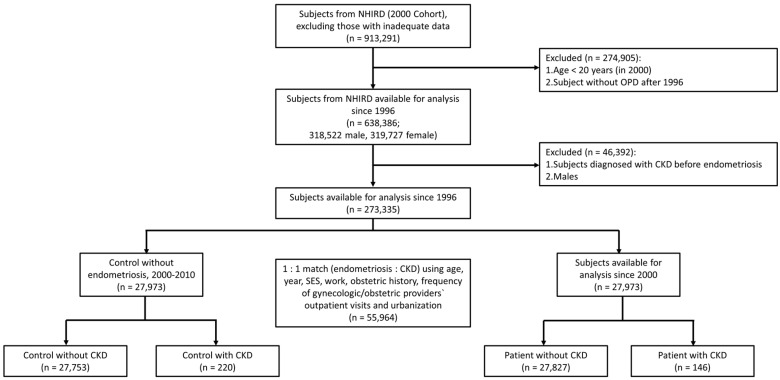
Cohort flow chart illustrating the inclusion and exclusion of participants in the study. NHIRD: National Health Insurance Research Database; OPD: outpatient clinics; CKD: chronic kidney disease.

This study which consisted of nearly the entire population of Taiwan (23 million inhabitants) was a retrospective cohort study approved by the Institutional Review Board of Taipei Veterans General Hospital (VGHIRB No.: 2012-12-012BC, 24 December 2015; the TPVGH-IRB (2) had reviewed and agreed to continue approving this trial and the approval date is extended from 24 January 2016 to 23 January 2017). The research database of Taiwan’s National Health Insurance (NHI) program from 1996 to 2010 contains 1 million randomly sampled beneficiaries (The Longitudinal Health Insurance Database 2000 (LHID 2000)). In the LHID 2000, the data of the sampled subjects are representative of all beneficiaries with regard to insurance cost, sex, and age, which have been previously described [41,42]. In Taiwan, the National Health Research Institute (NHRI) allowed access to the data in the National Health Insurance Research Database (NHIRD). During the study period, women without a visit to a gynecologist or obstetrician were excluded. The diagnostic criterion of women with endometriosis was based on at least 3 medical records (International Classification of Diseases, Ninth Revision, and Clinical Modifications (ICD9-CM) code 617) at outpatient clinics within 1 year or the mention of endometriosis by any doctor in at least 1 medical record from a hospitalization, which are frequently used criteria with which to diagnose women with endometriosis [9,41]; from 1 January 2000 to 31 December 2010, women meeting these criteria were included among the incident cases of endometriosis (*n* = 27,973), which yielded a prevalence of approximately 9%, which is similar to a report stating that women have a lifetime risk ranging from 4% to 15% [12]. To lower the influence of bilateral oophorectomy, bilateral salpingo-oophorectomy, and hysterectomy on the development of future CKD, women with hysterectomy were excluded, except those women with a diagnosis of CKD during the follow-up period. Each woman with endometriosis was matched with 1 woman control by age, frequency of gynecological/obstetric providers’ outpatient visits, obstetric history, index year, socioeconomic status, contraception methods, work, and urbanization. Thus, there was an overall sample of 27,579 matched controls without endometriosis (Figure 1).

The index date was the date of diagnosis of endometriosis for women with endometriosis. The index date was the date of admission or the first visit to an obstetric/gynecological provider during the study period for the controls. Initially, CKD was detected using inpatients with the ICD-9-CM 585 from the Registry for Catastrophic Illness Patients. Starting from the cohort index date, the study subjects were followed until hospitalization with CKD or until the end of the study (31 December 2010) if no CKD had occurred. Patients without a CKD event were treated as censored subjects. Those who were lost to follow-up or dropouts or were also treated as censored. Basic characteristics are presented as percentages. The incidence rate (IR) of CKD was compared between women with and without endometriosis.

### 4.2. Statistical Analysis

Among subsamples, the χ^2^ test was used to compare the IR estimates of CKD. The robust Cox proportional hazards model [43] was used to calculate the HR and 95% CI to determine whether newly diagnosed endometriosis is a risk factor for CKD. Adjusted variables in the Cox model were infertility status, pelvic inflammatory disease (PID), menopause, chronic liver disease, DM, CVD, and rheumatoid arthritis. All other data were analyzed using SAS version 9.3 (SAS Institute Inc., Cary, CA, USA), STATA version 10.0 (STATA Corp, College Station, TX, USA), and SPSS version 21 (IBM Inc., Armonk, NY, USA).

## 5. Conclusions

In the current study, women with endometriosis appeared to have a lower risk of CKD during the follow-up period compared with women without endometriosis; however, the protective role of endometriosis for CKD seemed to be less prominent after adjusting for menopausal status. Our findings should be confirmed in further studies.

## Figures and Tables

**Table 1:** Baseline characteristics of the study subjects.

Study or Subgroup	Total (*n* = 55,964)		Endometriosis (*n* = 27,973)		Controls (*n* = 27,973)		*p*-Value
Person-Years	628,337		314,514		313,823		
Variable	*n*	%	*n*	%	*n*	%	
Target							<0.0001
CKD	366	0.65	146	0.52	220	0.79	
No CKD	55,580	99.35	27,827	99.48	27,753	99.21	
Age *							0.6059
≤42	28,573	51.07	14,256	50.96	14,317	51.18	
>42	27,373	48.93	13,717	49.04	13,656	48.82	
SES							0.5376
≥40,000	6145	10.98	3059	10.94	3086	11.03	
20,000–39,999	13,382	23.92	6742	24.10	6640	23.74	
<20,000	24,582	43.94	12,313	44.02	12,269	43.86	
Others	11,837	21.16	5859	20.95	5978	21.37	
Work							0.7336
Yes	48,068	85.92	24,020	85.87	24,048	85.97	
No	7878	14.08	3953	14.13	3925	14.03	
Urbanization							0.8194
Urban	19,002	33.96	9475	33.87	9527	34.06	
Suburban	25,546	45.66	12,772	45.66	12,774	45.67	
Rural	11,398	20.37	5726	20.47	5672	20.28	
PID							<0.0001
Yes	37,928	67.79	22,449	80.25	15,479	55.34	
No	18,018	32.21	5524	19.75	12,494	44.66	
Infertility							<0.0001
Yes	3056	5.46	2356	8.42	700	2.50	
No	52,890	94.54	25,617	91.58	27,273	97.50	
Menopause							<0.0001
Yes	27,026	48.31	20,460	73.14	6566	23.47	
No	28,920	51.69	7513	26.86	21,407	76.53	
CVD							<0.0001
Yes	6411	11.46	3380	12.08	3031	10.84	
No	49,535	88.54	24,593	87.92	24,942	89.16	
DM							0.1343
Yes	7259	12.98	3689	13.19	3570	12.76	
No	48,687	87.02	24,284	86.81	24,403	87.24	
CLD							<0.0001
Yes	1851	3.31	1050	3.75	801	2.86	
No	54,095	96.69	26,923	96.25	27,172	97.14	
RD							<0.0001
Yes	2891	5.17	1672	5.98	1219	4.36	
No	53,055	94.83	26,301	94.02	26,754	95.64	

CKD: chronic kidney disease; SES: socio-economic status; PID: pelvic inflammatory disease; CVD: cardiovascular disease; DM: diabetes mellitus; CLD: chronic liver disease; RD: rheumatic disease. * Age variable was matched by the exact year of age, but the table shows age quartile groups. The median age of women with and without endometriosis was 40.5 and 40.4 years of age, respectively (*p* = 0.3570).

**Table 2:** Incidence and crude and adjusted risk of chronic kidney disease, according to endometriosis status.

Incidence or Risk	Patients with Endometriosis (*n* = 27,973)	Controls (*n* = 27,973)
Number of patients with CKD	146	220
Incidence per 10,000 person-years	4.64	7.01
Crude HR (95% CI)	0.65 (0.53–0.81) ***	1.00
Ad HR1 (95% CI)	0.69 (0.56–0.86) ***	1.00
Ad HR2 (95% CI)	0.85 (0.65–1.10)	1.00

CKD: chronic kidney disease; HR: hazard ratio; 95% CI: 95% confidence interval; Ad HR1: After adjustment of pelvic inflammatory diseases, infertility, cardiovascular disease, diabetes mellitus, chronic liver disease, and rheumatoid arthritis, we obtained the adjusted HR1 (Ad HR1); Ad HR2: After adjustment of pelvic inflammatory diseases, infertility, cardiovascular disease, diabetes mellitus, chronic liver disease, rheumatoid arthritis, and menopausal status, we obtained the adjusted HR2 (Ad HR2).***: *p* < 0.001.

**Table 3:** An increased risk of chronic kidney disease in women with endometriosis with age.

Age	<40 Years	40–49 Years	50–59 Years	60–69 Years	≥70 Years	*p* *
	*n* = 18	*n* = 55	*n* = 47	*n* = 20	*n* = 6	
IR	1.324	5.744	7.682	11.669	13.339	
C HR	1.00 (Ref.)	4.72 (2.74–8.13)	5.99 (3.44–10.44)	9.26 (4.85–17.68)	10.66 (4.20–27.06)	<0.0001
*p* **		<0.0001	<0.0001	<0.0001	<0.0001	
Ad HR1	1.00 (Ref.)	2.86 (1.58–5.16)	2.33 (1.24–4.37)	2.43 (1.16–5.07)	2.29 (0.84–6.23)	0.0162
*p* **		0.0005	0.0087	0.0181	0.1066	
Ad HR2	1.00 (Ref.)	2.92 (1.61–5.27)	2.53 (1.34–4.79)	2.64 (1.26–5.54)	2.46 (0.90–6.73)	0.0132
*p* **		0.0004	0.0044	0.0104	0.0803	

IR: incidence rate (incidence per 10,000 person-years); HR: hazard ratio; 95% CI: 95% confidence interval; Ref.: the youngest group (women < 40 years) as the reference; C HR: crude HR; Ad HR1: After adjustment of pelvic inflammatory diseases, infertility, cardiovascular disease, diabetes mellitus, chronic liver disease, and rheumatoid arthritis, we obtained the adjusted HR1 (Ad HR1); Ad HR2: After adjustment of pelvic inflammatory diseases, infertility, cardiovascular disease, diabetes mellitus, chronic liver disease, rheumatoid arthritis, and menopausal status, we obtained the adjusted HR2 (Ad HR2); *p* *: comparison among all groups; *p* **: comparison between study group and reference group (age <40 years); Data are presented as HR and (95% confidence interval).

**Table 4:** Incidence and crude and adjusted risk of chronic kidney disease, according to age.

Age	<40 Years (*n* = 23873)		40–49 Years (*n* = 18576)		50–59 Years (*n* = 10156)		60–69 Years (*n* = 2629)		≥70 Years (*n* = 712)	
	Patients	Controls	Patients	Controls	Patients	Controls	Patients	Controls	Patients	Controls
Diagnosis of CKD										
Yes	18	29	55	81	47	62	20	37	6	11
No	11872	11954	9310	9130	5001	5046	1298	1274	346	349
IR	1.32	2.12	5.74	8.53	7.68	10.17	11.67	21.97	13.34	24.18
Crude HR	0.59	1.00	0.67	1.00	0.73	1.00	0.53	1.00	0.55	1.00
(95% CI)	(0.32–1.07)		(0.48–0.95)		(0.50–1.07)		(0.31–0.92)		(0.20–1.50)	
Ad HR1	0.60	1.00	0.66	1.00	0.74	1.00	0.60	1.00	0.52	1.00
(95% CI)	(0.32–1.13)		(0.46–0.94)		(0.50–1.09)		(0.341–1.044)		(0.18–1.48)	
Ad HR2	0.68	1.00	0.85	1.00	1.06	1.00	0.62	1.00	0.94	1.00
(95% CI)	(0.29–1.61)		(0.57–1.25)		(0.65–1.73)		(0.30–1.25)		(0.18–4.93)	

Patients: women with endometriosis; Controls: women without endometriosis; CKD: chronic kidney disease; IR: incidence rate (incidence per 10,000 person-years); HR: hazard ratio; CI: confidence interval. Ad HR1: After adjustment of pelvic inflammatory diseases, infertility, cardiovascular disease, diabetes mellitus, chronic liver disease, and rheumatoid arthritis, we obtained the adjusted HR1 (Ad HR1); Ad HR2: After adjustment of pelvic inflammatory diseases, infertility, cardiovascular disease, diabetes mellitus, chronic liver disease, rheumatoid arthritis, and menopausal status, we obtained the adjusted HR2 (Ad HR2).

**Table 5:** Age and surveillance time between enrollment in the cohort and the diagnosis of chronic kidney disease.

CKD	Total (*n* = 366)	Endometrioses (*n* = 146)	Controls (*n* = 220)	*p*-Value
Age (years)				
Mean ± SD	50.6 ± 11.1	50.2 ± 10.8	50.8 ± 11.4	0.6367
Median (Min–Max)	49.5 (19–82)	49.5 (19–81)	49.5 (20–82)	
Interval (years)				
Mean ± SD	6.53 ± 4.50	7.58 ± 4.03	5.83 ± 4.67	0.0002
Median (Min–Max)	6.47 (0–15.29)	7.45 (0.13–15.29)	5.86 (0–14.97)	

Age: age at the diagnosis of chronic kidney disease; SD: standard deviation; Interval: interval between enrollment in the cohort and the diagnosis of chronic kidney disease; Min: minimum; Max: maximum.

**Table 6:** Age and surveillance time between enrollment in the cohort and the end of the last follow-up.

All Women	Total (*n* = 55,946)	Endometrioses (*n* = 27,973)	Controls (*n* = 27,973)	*p*-Value
Age (years)				
Mean ± SD	41.6 ± 11.5	41.6 ± 11.5	41.6 ± 11.6	0.8205
Median (Min–Max)	42.0 (16–97)	42.0 (16–94)	42.0 (16–97)	
Interval (years)				
Mean ± SD	11.231 ± 4.11	11.24 ± 4.10	11.22 ± 4.12	0.4780
Median (Min–Max)	12.91 (0–15.29)	12.91 (0.01–15.29)	12.92 (0–15.16)	

Age: age at the time of enrollment; SD: standard deviation; Interval: interval between enrollment in the cohort and the end of the last follow-up; Min: minimum; Max: maximum.

## Abbreviations

MDPI	Multidisciplinary Digital Publishing Institute
DOAJ	Directory of Open Access Journals
CKD	chronic kidney disease
HR	hazard ratio
IR	incidence rate
CI	confidence interval
GFR	glomerular filtration rate
ESRD	end-stage renal disease
DM	diabetes mellitus
CVD	cardiovascular disease
NHI	National Health Insurance
LHID	Longitudinal Health Insurance Database
NHRI	National Health Research Institute
NHIRD	National Health Insurance Research Database
ICD9-CM	International Classification of Diseases, Ninth Revision, and Clinical Modifications
PID	pelvic inflammatory disease
OR	odds ratio
ER	estrogen receptor
GPER	G-protein-couple protein 30
ERK	elementary response kinase
OPD	outpatient clinics
TPVGH-IRB (2)	Institutional Review Broad (2) of Taipei Veterans General Hospital
